# Spontaneous Intracranial Hypotension and Subdural Hematomas Treatment Management Using MMA Embolization and Target Blood Patch: A Case Report

**DOI:** 10.3390/life14020250

**Published:** 2024-02-13

**Authors:** Luigi Cirillo, Francesca Verna, Ciro Princiotta, Massimo Dall’Olio, Arianna Rustici, Carlo Bortolotti, Filippo Badaloni, Davide Mascarella, Pietro Cortelli, Sabina Cevoli

**Affiliations:** 1 UO Neuroradiologia, IRCCS Istituto delle Scienze Neurologiche di Bologna, 40139 Bologna, Italy; luigi.cirillo2@unibo.it (L.C.); c.princiotta@isnb.it (C.P.); m.dallolio@isnb.it (M.D.); 2 Dipartimento di Scienze Biomediche e Neuromotorie (DIBINEM), Alma Mater Studiorum University of Bologna, 40126 Bologna, Italy; arianna.rustici2@unibo.it; 3 UO Neurochirurgia, IRCCS Istituto delle Scienze Neurologiche di Bologna, 40139 Bologna, Italy; bortolotticarlo@yahoo.it (C.B.); fbadaloni@gmail.com (F.B.); 4 UO Clinica Neurologica Metropolitana (NEURO-MET), IRCCS Istituto delle Scienze Neurologiche di Bologna, 40139 Bologna, Italy; davide.mascarella2@unibo.it (D.M.); pietro.cortelli@unibo.it (P.C.); sabina.cevoli@unibo.it (S.C.)

**Keywords:** spontaneous intracranial hypotension, subdural hematoma, blood patch, headache, middle meningeal artery embolization

## Abstract

We report a patient suffering from spontaneous intracranial hypotension (SIH) who, following a non-selective lumbar blood patch, returned to his healthcare provider with severe symptoms of neurological deficits. It was subsequently discovered that the aforementioned deficits were due to a bilateral subdural hematoma, and an emergency surgical drainage of the hematoma has been performed. However, the hematoma reformed and potential cerebrospinal fluid leakage was consequently investigated through myelography. Following the diagnostic finding of a venous diverticulum, a selective blood patch was executed in the affected area, and in order to stabilize the hematoma, an embolization of the middle meningeal arteries was performed. The combination of such operations allowed for the resorption of the hematoma and the improvement of neurological symptoms.

## 1. Introduction

Spontaneous intracranial hypotension (SIH) is a pathological condition characterized by an orthostatic headache and low cerebrospinal fluid (CSF) pressure, resulting from the spontaneous leakage of CSF at the spinal level [1]. The certain cause is unknown to date. The primary clinical condition is characterized by orthostatic headaches, often experienced during the latter half of the day. Patients may also report auditory symptoms, such as tinnitus or muffled hearing. Additional common accompanying symptoms are phonophobia, photophobia, dizziness, and nausea [2,3,4]. Clinical symptoms are frequently associated with a wide range of radiological brain images. Specifically, they may include a diffuse pachimeningeal enhancement, the downward displacement of the midline structures with brainstem sagging, venous sinus expansions, and subdural collections, which may consist of CSF or blood [5,6]. Voluminous subdural blood collections are rare consequences of SIH, and to the best of our knowledge, an accepted guideline for its optimal management is not reported at date. Many SIH patients recover after conservative treatments, such as hydration, rest, and caffeine; further cases required invasive treatments, such as the application of epidural blood patches (EBP) [7]. Blood patch treatments can be both non-selective in the lumbar region or specific at the leakage point: usually a non-selective treatment is employed as a first-line approach, and hence, the leakage site investigation through spine MRI it is not required. Targeted patches should be performed in patients who remain symptomatic following appropriate conservative management and/or non-targeted EBPs, in whom a causative lesion has been identified on DSM or CTM, which is safely accessible via an image-guided transcutaneous approach. [8]. In the latter case, the leakage point is identified through diagnostic methods, such as spine MRI, myelography, or myelo-CT [9]. In some rare instances, SIH patients may experience a subdural hematoma (SDH) as uncommon complication, which can lead to severe neurological deficits [10]. In similar cases, the embolization of the middle meningeal artery could be a valid approach since it enhances the complete reabsorption of hematomas [11].

## 2. Case Report

**Figure 1:**
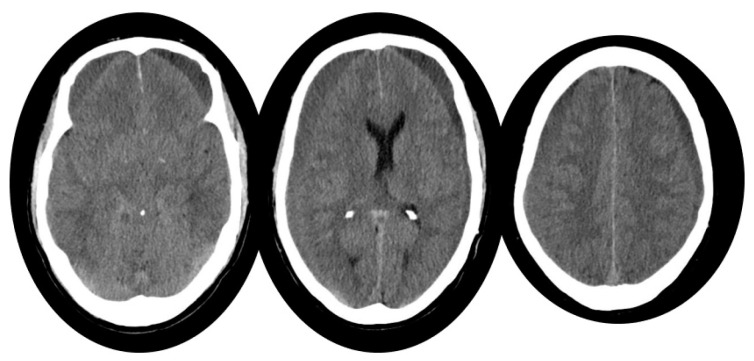
Brain CT showing bilateral subdural hematomas (SDH) without suffering of brain tissue.

A 59-year-old male patient presented with a recent history of cervical pain, orthostatic headache, hearing disturbances, and cognitive slowing, not significantly relieved by conservative therapy. The anamnestic evaluation uncovered a ski-related fall one month before onset. Considering the patient’s neck pain, it was decided to investigate the symptom with a cervical MRI. The cervical magnetic resonance revealed notable cervical osteophyte bars compressing the dural sac, but no signs of cervical trauma were evident. A subsequent brain computed tomography (CT) showed recent bilateral subdural hematomas (SDH) and the classic sign of SIH (diffuse pachimeningeal enhancement, downward displacement of the midline structures with brainstem sagging, venous sinus expansions, and subdural collections). Accordingly, he was diagnosed with SIH (BERN SCORE 9), complicated by bilateral subdural hematoma, above and below the tentorium (Figure 1).

The patient was accordingly treated with a first-level therapy approach: non-selective lumbar epidural blood patch, which was well tolerated with mild post-procedural orthostatic headache improvement, the patient was discharged three days after the procedure. However, three weeks after the aforementioned treatment, the patient reported multiple episodes of headaches, confusion, spatial–temporal disorientation, incongruent behaviors, dysphagia, and projectile vomiting in the ensuing weeks. A brain CT scan revealed an increase in the subdural hematoma, especially in the right side. The SDH displaced the brain structures towards the left (approximately 7 mm), with a slight enlargement of the temporal horns and a downward displacement of midline structures.

**Figure 2:**
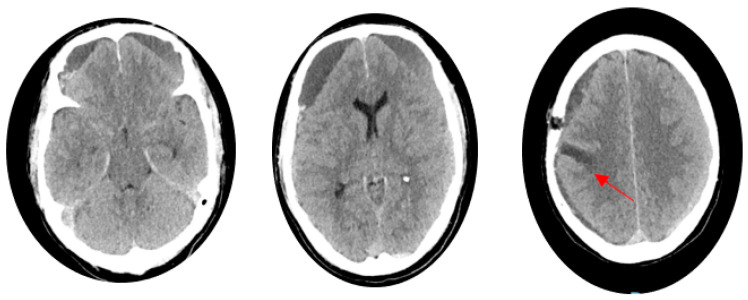
Post-surgery brain CT showing the surgical entry hole and an area of slight tissue suffering in the right post-Rolandic site (red arrow).

The patient was then admitted to neurosurgery, presenting closed eyes, regular arousal, cooperation, and orientation in space and time, with no focal deficits (Glasgow Coma Scale 14/15). During the night, the patient was found on the floor due to a suspected fall (GCS 12/15). The occurrence required an urgent brain CT scan, which revealed the partial rebleeding of the previously identified subdural collections. Therefore, an emergency evacuation of the right-sided subdural collection was performed (Figure 2). Afterwords, the patient denied any headache and other significant symptoms (GCS 15/15); therefore, he was discharged. 

**Figure 3:**
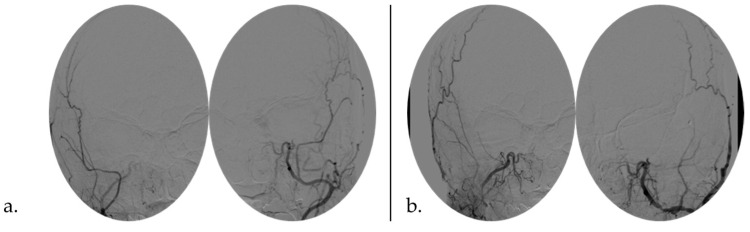
Angiographic detection (**a**) pre- and (**b**) post-MMA embolization.

Four days after, the patient has once again experienced headaches, projectile vomiting, and fatigue. A brain CT scan revealed a further increase in the already reported right-sided subdural collection. The patient appeared confused, and two episodes of urinary incontinence occurred. A subsequent emergency brain CT scan demonstrated an enlargement of subdural collections in the frontal region, especially on the left side. Considering the CT findings and the evolving clinical presentation, a unilateral surgery evacuation of the subdural hematoma was deemed necessary, performed, and followed by the endovascular embolization of MMA to avoid rebleeding. The endovascular treatment was performed by bilaterally injecting 300-micron particles in the distal frontal and parietal branches. At the end, coils in proximal common branches of MMA were injected to allow for a more stable occlusion of the proximal vessel and to improve the effectiveness of the treatment [12] (Figure 3).

In the following two days, the patient developed ideomotor deceleration, speech difficulties, and fatuous behavior but without any headache complaints. Dysphagia was also observed, leading doctors to prescribe a diet with modified consistency and thickened fluids. Brain and spinal MRI did not reveal acute brain lesions but showed a minimal increase in the thickness of the subdural hematoma collection along the hemispheric convexity, particularly on the right side. Meningeal thickening emerged, predominantly on the right side in the anterolateral region after the application of contrast. An anterior epidural collection in C2–C6 region was eventually observed. Since the MMA embolization had halted the expansion of the hematoma’s collection, it was decided to perform a targeted blood patch. Before performing the blood patch treatment, it is necessary to identify the site of the leakage.

For this reason, the embolization was followed, three days later, by a second-level diagnostic/therapeutic approach, dynamic myelography, in order to document any cerebrospinal fluid leakage. After the procedure, the patient did not exhibit neurological disturbances and did not report headaches.

**Figure 4:**
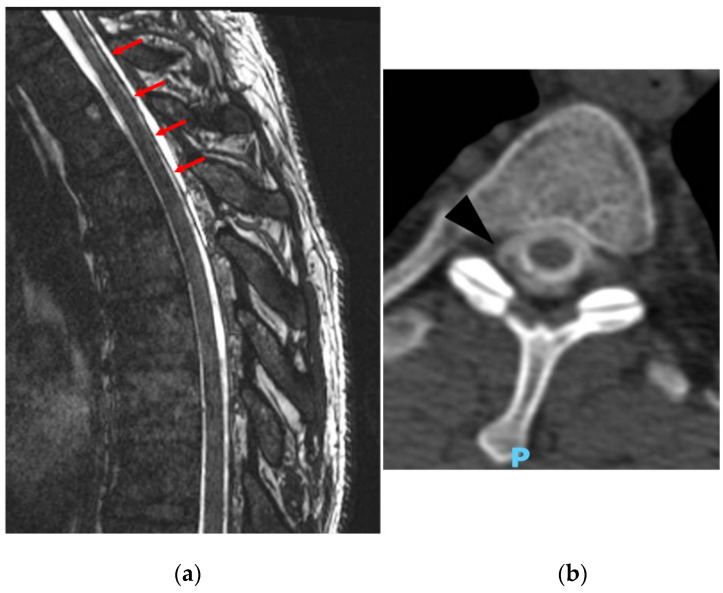
(**a**) Sagittal view of 3T MRI cisternographic sequence demonstrating posterior epidural collections in the cervical–dorsal tract (red arrows). (**b**) Axial reconstruction of Myelography CT showing the epidural contrast medium leakage (black arrow) at right side of D2–D3 level (Type II of epidural leake).

The myelography examination indicated an increased representation of the dural sac at the cervical–dorsal transition, proving the presence of multiple lateral meningeal diverticula and a very thin postero-lateral leak in proximal dorsal region. The myelography was followed by a myelographic CT, which is a diagnostic procedure applied to confirm the dorsal leakage site of CSF (which is displayed as an epidural spread of the contrast medium), which has been observed in the myelography previously performed. Therefore, an epidural spread of the contrast medium was demonstrated and confirmed in the myelographic CT (Figure 4).

**Figure 5:**
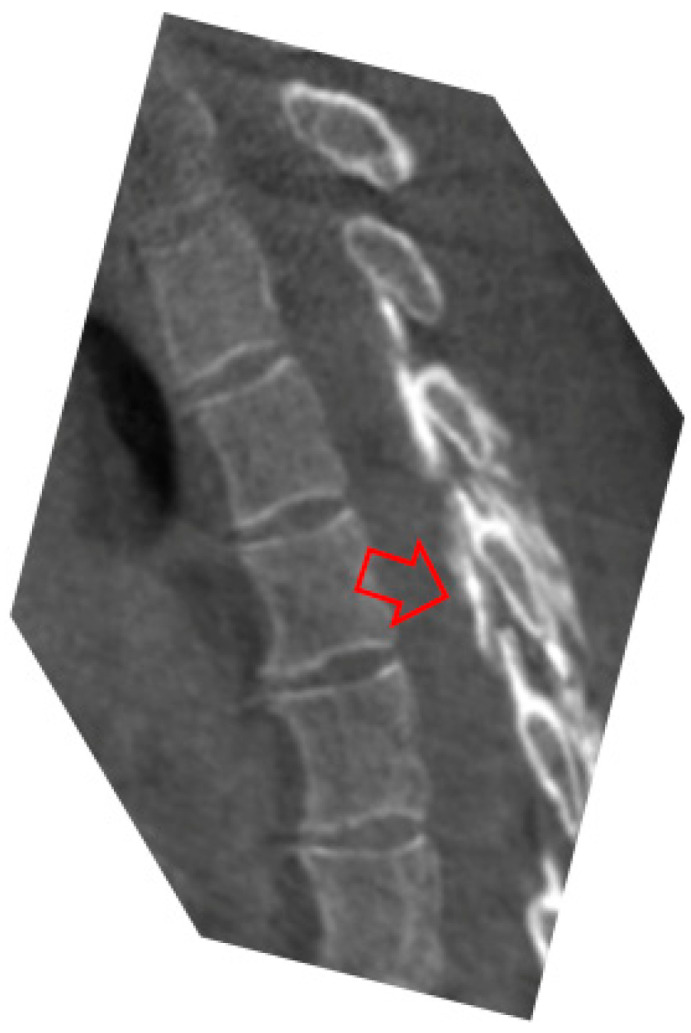
Post-blood patch dynaCT XperCT control with Cone beam technique confirms the amount of contrast at the epidural site (red arrow).

The patient underwent a targeted blood patch procedure, with access at D2–D3 and D3–D4 interspinous spaces under fluoroscopic guidance. The blood patch was performed with an injection of approximately 25 cc of autologous blood. (Figure 5).

The patient tolerated the procedure well, without an exacerbation of pain. In the following two days, the patient’s cognitive abilities improved.

An evaluation of headaches and frontal function was conducted using the FAB scale.

The FAB scale is a brief screening test consisting of six subtests to assess various abilities controlled by the frontal lobes: classification, mental flexibility, motor programming, interference sensitivity, inhibitory control, and environmental autonomy. Each of the various subtests is associated with a score. The maximum score achievable by the patient is 18 points [13]. In this case, it has been decided to administer the FAB test at three different time points:

T0: It is time zero, the first measurement of the test, with the patient in a supine position.
T1: It is the second measurement of the test after the patient has been in an upright position for 5 min.
T2: It is the third measurement of the test after the patient has been in an upright position for 20 min.

It has also been decided to assess how the patient’s orthostatic headache evolves over the various time points. The results were as follows:

T0: Headache NRS 2–3/10, with only slight occipital discomfort. FAB score 17/18, losing one point in the grip test. T1: Stable headache. FAB score 16/17, losing one point in the grip test and one point in verbal fluency. T2: Stable headache. FAB score 15/17, losing one point in the grip test, one point in verbal fluency, and one point in the Go-no-go test.

The test was conducted because the patient, due to hematomas, exhibited a deficit in cognitive abilities. Therefore, it was important to assess whether there was an improvement in these abilities over time.

Moreover, since SIH (spontaneous intracranial hypotension) is a condition that predominantly manifests symptoms (such as headaches) in an upright position, it was decided to conduct the tests at three different time points to monitor the evolution of symptoms from a supine position to an upright position.

To resume, the patient underwent four invasive treatments: first, an epidural nonselective blood patch was performed due to the SIH diagnosis (i). Then, when the subdural hematoma has shown signs of expansion, an emergency evacuation of the right-sided subdural collection was accomplished (ii). Despite the surgical treatment, a few days later, the patient still showed signs of hematoma expansion, so it was decided to perform the MMA embolization (iii). At the end, after the myelographic examination showed the presence of a leakage, the patient underwent a selective epidural blood patch (iv).

**Figure 6:**
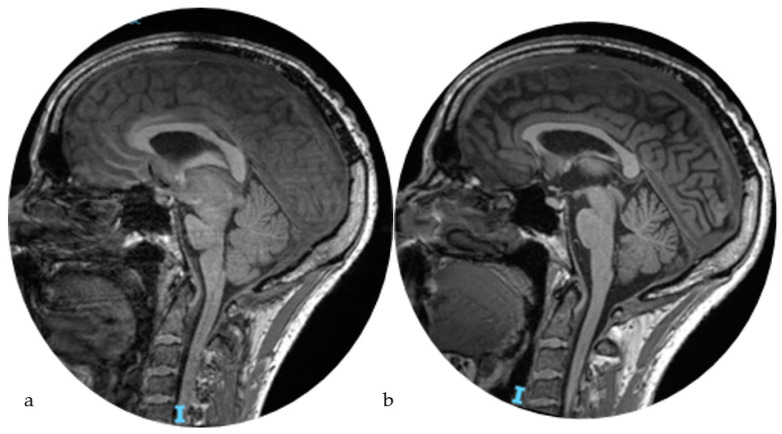
Brain MRI pre-procedures (**a**) and two months after (**b**) showing the restitutio ad integrum of brain sagging.

In the subsequent days, the patient underwent a rehabilitation program for orthostatic retraining and dysphagia. The patient was finally discharged in a stable clinical condition, free of headaches, and self-contained. During the successive follow-up visits for up to 12 months, the patient remained asymptomatic, and MRI examinations presented the complete reabsorption of the subdural collections and the improvements of the neuroradiological profile (Figure 6).

## 3. Discussion

The paper proposes an overview of the clinical signs and symptoms that characterized the reported clinical situations, with the aim to enhance the knowledge about the disease and enrich the currently available literature related to the topic. An improvement of the knowledge and of the available information would reduce the episodes of SIH misdiagnosis, which is recognized to imply a consequent development of SDH, probably because of a delay in the correct diagnosis of SIH [14].

SIH is caused by low CSF pressure, usually secondary to an occult leak. A CSF leak occurs in weak areas around the dura mater and nerve root sheaths and around small defects due to small traumas, a fall, severe exercise, or a cough that tears the dura or arachnoid [15]. The primary goal of SIH treatment is to stop the CSF leakage and to increase the CSF volume. Traditionally, treatment for SIH begins with conservative therapy, including hydration, analgesics, and abdominal binders. Autologous EBP is considered the preferable treatment for patients who have failed initial conservative treatments [16]. Although nontargeted epidural blood patching is often used as a first choice to treat SIH, it may not provide durable relief in a significant number of patients. For patients for whom nontargeted patching fails, targeted epidural patching or surgical intervention may be necessary. In the latter situation, the precise location of the site of the leak must be identified [9]. There is evidence that, by increasing the proximity of the targeted therapy to the site of the CSF leak, the outcomes can be improved. Therefore, diagnostic invasive techniques that precisely localize a CSF leak are essential to properly guide the therapy. CT myelography (CTM) is probably the most common strategy to investigate CSF leaks. In fact, it is considered by many authors to be the preferable diagnostic examination to refer to in the case of suspected SIH. In CTM, the introduction of an iodinated contrast material into the thecal sac allows for the adequate visualization of CSF, including CSF that has leaked into the epidural space [9]. As demonstrated by the above-mentioned case study, it can be affirmed that the diagnosis of SIH should always be followed by an invasive diagnostic investigation, such as CTM, which often allows for the identification of the leakage and subsequently targeted patch treatment. In a significant proportion of cases of SIH, as well as in our case, no precise leaks were detected on myelography despite an exhaustive investigation, and no precise leakage points were identified. However, meningeal diverticula were pointed out. Regardless of the lack of knowledge of the specific leakage site, empirical patching targeted at diverticula may be efficacious in these patients, although it becomes more technically challenging as the number of diverticula increases [17]. Hygromatous subdural collections are frequently detected in patients diagnosed with SIH, varying in appearance from thin to larger subdural hygromas with significant mass effect [10]. Subdural hematoma is a serious complication of SIH. While the pathophysiology of SDH in patients with SIH remains unknown, studies have proposed several mechanisms. The downward displacement of the brain due to low CSF pressure may produce tears in the bridging veins of the dural border cell layer, causing these veins to rupture. Alternatively, as subdural CSF collections gradually enlarge the subdural space, the bridging veins may stretch and rupture in some cases [15]. If the presence and the growth of the SDH is not immediately treated, it may lead to an important mass effect with severe neurological symptoms. The discussed clinical case represents a valid example since the development of SDH implied an urgent and complex surgical drainage of the hematoma, risking the patient’s life. The surgical drainage of SDH associated with SIH is particularly dangerous due to the high probability of incurring development episodes in relapses. However, the diagnostic approach of SIH requires a considerable period of time, and in order to facilitate its management, the embolization of the arachnoid membrane (MM) can be considered a valid and safe treatment. It is necessary to avoid the rapid growth of hematomas, especially in young patients with a poor ability to tolerate the mass effect. A valid choice to promote the reabsorption may be an endovascular treatment with the embolization of the middle meningeal artery (MMA), an emerging minimally invasive endovascular technique for chronic subdural hematoma (cSDH) [18]. The aim of the procedure is to interrupt the “refilling” mechanism of the hematoma by bilaterally interrupting the blood supply from the middle meningeal artery to the dura mater: it is widely known that subdural hematomas arise from the rupture of bridging veins [19]. The blood from the ruptured vein and cerebrospinal fluid beneath the dura mater merge together: this results in the gradual accumulation of bloody fluid, coating the cavity and leading to hematoma formation.

Histopathological examination reveals a two- to three-layered structure in the membranes of the hematoma cavity. The inner layer presents small neovessels. The mechanism driving hematoma cavity enlargement is believed to involve intermittent bleeding into the hematoma cavity due to the rupture of these neovessels [20].

In additional research, communicating vessels between the dura mater and the membranes of the subdural hematoma were identified [21]. These vessels, classified as capillary-like vessels, small veins, and small arteries, penetrate through the dura mater and connect to the middle meningeal artery (MMA). The MMA, which provides a blood supply to the dura mater, is believed to give rise to the capillary feeders that supply the cSDH [22]. This finding indicates that the MMA embolization has the potential to interrupt the blood supply to the hematoma.

The above-mentioned case report suggests that MMA embolization, applied as the primary approach for patients with subdural hematoma, enhances treatment outcomes and decreases the need for surgical rescue with a low risk of morbidity and mortality [23].

## 4. Conclusions

We can assert that, in a patient with SIH, it is crucial to investigate the site of cerebrospinal fluid leakage as the primary diagnostic approach using invasive techniques such as myelography and myelo-CT. In the event that these techniques do not identify the site of leakage with adequate accuracy, a selective blood patch at the dural leak site or at the mayor dural tears is recommended.

If a patient with diagnosed SIH also presents a subdural hematoma, prompt action is necessary to prevent mass effect symptoms that can result in significant neurological deficits. In that case, the embolization of the middle meningeal arteries can be suggested as a valid technique. This procedure allows for the interruption of the arterial blood refill of the dura membrane/pseudomembrane, preventing the hematoma rebleeding and a serious post-surgical complication, accelerating the reabsorption. This approach may avoid highly invasive and risky surgical interventions, such as the surgical drainage of the hematoma. According to previous studies [24], MMA embolization represents an innovative and largely successful emerging treatment modality.

## Data Availability

Data will be available under request to the corresponding author: francesca.verna2@studio.unibo.it.
